# The Clinical Outcomes of Operative Treatment Versus Conservative Treatment for Dancer’s Fractures: Protocol for a Retrospective Cohort Study

**DOI:** 10.2196/37171

**Published:** 2022-04-05

**Authors:** Marinus Alexander de Ruijter, Jian Zhang Yuan, Robert Jan Derksen

**Affiliations:** 1 Department of Traumasurgery Zaandam Medical Center Zaandam Netherlands

**Keywords:** Dancer’s fracture, fifth metatarsal fracture, outcomes, surgery, nonoperative treatment

## Abstract

**Background:**

Fifth metatarsal fractures are one of the most common foot fractures, and 11% to 25% of such fractures are Dancer’s fractures (distal spiral fractures). Conservative therapy while wearing a cast and operative treatment have been used as preferred modes of treatment in the limited literature available. However, we often see healing problems, such as delayed union and nonunion, when Dancer’s fractures are treated nonoperatively, resulting in a need for secondary intervention. In our institution, treatment has changed over the years from predominantly conservative treatment to mostly operative treatment. To investigate whether our hypothesis holds true that primary surgical treatment is beneficial, a retrospective study was designed.

**Objective:**

The objective of the study is to compare differences between outcomes (delayed union and nonunion) of conservative and operative treatments for Dancer’s fractures.

**Methods:**

A retrospective comparative cohort study will be conducted in a level II trauma center (Zaandam Medical Center). Patients who experienced a Dancer’s fracture in the period of 2012 to 2021 will be included and divided into 2 cohorts—the conservative (2012-2015) and operative (2016-2021) treatment cohorts. The primary outcome will be the differences in percentages of delayed union and nonunion between the two groups. The secondary outcomes will be the percentage of primary conservative treatment failure, the need for secondary operative treatment, complications (infection and hardware failure), and functional outcomes. If 118 patients are included in each group, sufficient power is expected to be reached, depending on the age distribution of patients. The percentages of delayed union and nonunion among the two groups will be calculated and statistically compared via chi-square statistics. A logistic regression analysis will be used to investigate possible associations between patient characteristics and failed conservative treatment. A Mann-Whitney *U* test will be used to compare functional outcomes between groups. An independent, 2-tailed *t* test will be used to compare mean 12-Item Short Form Survey scores if they are normally distributed, and a Wilcoxon rank sum test will be used if they are nonnormally distributed.

**Results:**

In total, 2134 potentially relevant health insurance codes have been extracted from the hospital’s register. We expect to find a total of 236 Dancer’s fractures in this data set.

**Conclusions:**

Our study has limitations due to it being a single-center study and data collection being performed retrospectively. However, it covers a large time period and may provide the possibility to show treatment outcome differences (delayed union and nonunion, complications, and functional outcomes) in 2 reasonably large cohorts (conservative and operative treatment cohorts), which has not been done before in literature on Dancer’s fractures. If our hypothesis that surgery is beneficial for Dancer’s fractures is proven true by our study, we plan to further corroborate it by conducting a prospective randomized controlled trial.

**International Registered Report Identifier (IRRID):**

PRR1-10.2196/37171

## Introduction

Fifth metatarsal fractures are one of the most common foot fractures [1,2]. There is no unified classification system; however, the Lawrence and Botte classification is recommended [3]. The fifth metatarsal bone is divided into 3 anatomical zones where fractures can occur. Zone 1 is the tuberosity; zone 2 is the metaphyseal-diaphyseal junction, which extends into the fourth-fifth intermetatarsal facet and is also known as the *Jones fracture*; and zone 3 consists of the proximal diaphyseal fractures, which are located within 1.5 cm of the tuberosity [4]. Another type of fracture is the so-called *Dancer’s fracture*—a long spiral fracture that extends into the distal metaphyseal area [4]. Dancer’s fractures are diagnosed in 11% to 25% of fifth metatarsal fractures [1,5,6] and 5% of metatarsal fractures overall [7]. The optimal treatment for this fracture type is still under debate, and most of the available related literature only consists of small retrospective studies or case series.

Studies have shown different outcomes of treatments. Some have used conservative therapy with or without weight-bearing immobilization for 6 to 8 weeks and achieved excellent results [6-10]. Others have used surgical therapy with plates and screws, which resulted in excellent outcomes [11,12] that were comparable to those of the same therapy for shaft fractures of other metatarsal bones [13]. Patient characteristics such as age and osteoporosis [5,14], as well as fracture characteristics such as the angulation of the fracture and comminution of the fragments, are important factors in the choice of treatment [15]. Dancer’s fractures are usually angulated and short [11]. It has been shown previously that dislocation is associated with functional impairment in patients with metatarsal fractures [2]. Therefore, malunion, delayed union, or nonunion is expected if Dancer’s fractures are treated nonoperatively. Due to advancing insights, from 2016 onward, the preferred treatment in our hospital changed from predominantly conservative treatment to mostly operative treatment. Our hypothesis is that Dancer’s fractures often result in healing problems, such as delayed union and nonunion, when treated nonoperatively.

The aim of our study is to compare outcomes of primary operative treatment for Dancer’s fractures to those of conservative treatment. It is hypothesized that primary operative treatment can be beneficial when it comes to reducing delayed union and nonunion rates.

## Methods

### Study Objectives

The primary objective is to provide an overview of all Dancer’s fractures, the applied initial treatments, and final outcomes—delayed union and nonunion rates and complications. The secondary objective is to determine how many fractures needed surgery after they were initially treated conservatively. The third objective is to identify associations between patient characteristics and failed initial conservative treatment. Finally, we will measure functional outcomes in both patient groups.

### Study Design and Setting

Our study will be a retrospective cohort study. The researchers will be provided with records and diagnostic health insurance codes by the hospital’s medical administration. These codes represent patients with foot injuries (codes 237 and 238 for metatarsal and tarsal fractures, respectively) over the past 10 years in a single, regional, level II trauma center—the Zaandam Medical Center, Zaandam. These cases will be retrospectively analyzed to select oblique diaphyseal fractures of the fifth metatarsal bone, which are known as *Dancer’s fractures*. Since 2016, in our hospital, the preferred treatment changed from predominantly conservative treatment to mostly operative treatment. Therefore, the comparison of 2 historic cohorts will be made—the conservative treatment cohort (2012-2015) versus the operative treatment cohort (2016-2021).

### Study Population

Patients aged ≥18 years who presented to the emergency department or outpatient clinic with a Dancer’s fracture within 48 hours after trauma will be considered eligible for participation in our study.

### Exclusion Criteria

Patients with multiple, simultaneous ipsilateral foot or ankle fractures; open fractures; known, pre-existing, significant impaired mobility; or any pretrauma, gross anatomical anomaly of the foot will be excluded from participation in our study.

### Recruitment

A list of all foot fracture–related diagnostic health insurance codes in the Zaandam Medical Center dating from 2012 to 2021 will be provided by the hospital’s medical administration. All corresponding radiographs of the foot will be reviewed by 2 trauma surgeons to identify Dancer’s fractures. Patient characteristics such as age and sex, as well as fracture characteristics such as comminution, dislocation, the side of injury, and the occurrence of delayed union and nonunion, will be extracted from the electronic patient records manually by the researcher. Delayed union and nonunion are defined as absent or incomplete fracture healing after 3 and 6 months, respectively, and they will be assessed by reviewing the radiographs. The given treatments (cast vs surgery) and occurrence of complications (infection and hardware failure) will also be extracted from the electronic patient records manually by the researcher. The cohort will be divided into the following two groups: the conservative treatment cohort (2012-2015) and operative treatment cohort (2016-2021). Patients will receive a letter about providing informed consent. The letter will state that after they provide consent, a researcher will call them. Patients will be asked if they underwent secondary surgery in another hospital and be asked to provide data that are missing from the electronic patient files, such as the mechanism of injury and smoking status at the time of injury.

Patients will also be asked to complete the 12-Item Short Form Survey (SF-12) questionnaire and to rate their functional outcomes by using 1 of the following 3 categories: (1) no more complaints or pain, (2) minor complaints or pain without an impact on daily living, and (3) significant complaints or pain with an impact on daily living [16].

If patients are lost to follow up, it will be assumed that they did not experience delayed union, nonunion, or other complications.

### Outcomes

The primary outcome will be the delayed union and nonunion rates in the primary operative treatment group versus those in the conservative treatment group. The secondary outcomes will be the percentage of initial conservative treatment failure, which will be determined based on the percentage of patients who needed surgery after initially being treated conservatively; the number of patients who experience complications (infection and hardware failure); and associations between patient characteristics and failed initial conservative treatment. Functional outcomes—the complaints and impacts on daily living that are reported and experienced by patients—will also be secondary outcomes. These will be measured based on patients’ responses to the SF-12 and 3 categories (no more complaints or pain, minor complaints or pain without an impact on daily living, and significant complaints or pain with an impact on daily living).

### Sample Size

Based on the limited available literature, delayed union and nonunion have been found in 3% to 9% [7,8] of patients in the general population who were treated nonoperatively. For patients aged ≥40 years, the delayed union rate for nonsurgical treatment is 35% [5]. This rate ranges from 4% to 7% for patients who were treated operatively [11,12]. It is expected that 236 patients will be included in our study, with approximately 118 in each cohort. Based on a post hoc power analysis, a power of 34.3% can be achieved by recruiting patients from the general population, and a power of 100% can be achieved by recruiting patients aged ≥40 years. As the incidence rates of delayed union and nonunion are unreliable in the available (limited) literature, the post hoc power analysis showed large differences in power—a problem that is often encountered [17]. However, our cohort will be the largest cohort of patients with Dancer’s fractures to date. Based on our clinical experience, we also believe that the incidence of delayed union and nonunion might be high in the nonoperative treatment cohort. The data collected in this study can be used to calculate the number of patients that need to be included in future prospective studies.

### Statistical Analysis

Data will be processed and analyzed by using IBM SPSS Statistics version 25 (IBM Corporation). Descriptive data will be shown as numbers, percentages, or means with SDs when the data are normally distributed and as medians with IQRs when the data are nonnormally distributed. The percentages of delayed union and nonunion among the two groups will be calculated and statistically compared by using chi-square statistics. A multiple regression analysis will be used to investigate possible associations between patient characteristics and failed conservative treatment. A Mann-Whitney *U* test will be used to compare functional outcomes between groups. An independent, 2-tailed *t* test will be used to compare mean SF-12 scores if they are normally distributed, and a Wilcoxon rank sum test will be used if they are nonnormally distributed.

### Ethical Considerations

All collected data will be processed and filed anonymously on the hospital’s secured servers. Data will be stored in separate files. One file will contain coded patient information, and a second file will contain all variables and diagnoses that are linked to these codes. The key to this code will be safeguarded by the head researcher. Patients will be asked via mail to provide informed consent prior to being contacted by telephone. The study is not subject to the Medical Research Involving Human Subjects Act (*Wet medisch-wetenschappelijk onderzoek met mensen* [WMO]). This Dutch act states that official medical ethics approval is not needed as long as patients are not subjected to invasive maneuvers, a specific code of conduct is used, and questionnaires do not contain sensitive questions (eg, those concerning mental health status) [18,19]. Confirmation of this status (*niet-WMO-verklaring*) will be obtained from the regional medical ethics committee.

## Results

Approximately 2667 potentially relevant health insurance codes from a period of 10 years (2012-2021) will be extracted from the hospital’s digital register. To date, 2134 codes have been extracted. We expect to find a total of 236 Dancer’s fractures, of which approximately 50% (n=118) have been intentionally treated conservatively and 50% (n=118) have been primarily treated operatively. Based on our hypothesis and daily practice experience, it is expected that delayed union and nonunion rates will be higher in the conservative treatment group.

## Discussion

### Principal Results

We expect to find a total of 236 cases that are eligible for inclusion. Considering the type of injury and available literature, ours may be one of the largest cohorts for reviewing surgical treatment so far. It is hypothesized that the rates of delayed union and nonunion will be higher in the nonoperative treatment group. We may find that impacts on daily living are associated with more complaints and lower SF-12 scores and that a significant percentage of patients needed to undergo secondary surgery after conservative treatment. In concordance with previous literature, low postoperative infection rates and low rates of hardware failure are expected.

### Comparison With Prior Work

Previous studies have looked at either nonoperative treatment [6-10] or operative treatment [11,12]. To our knowledge, no studies have compared operative treatment to nonoperative treatment before. We expect to find higher rates of delayed union and nonunion in older, female patients [5] and those who smoke, similar to previous findings in related literature. Patients with displaced fractures might benefit the most from surgical intervention, as suggested by Thompson et al [11].

Considering the knowledge gap in literature with regard to the optimal treatment of Dancer’s fractures, it is important to first gain insight into our hypothesis before applying for grants for a costly randomized controlled trial.

### Strengths and Limitations

Several limitations apply to our study. Since ours is a single-center study, included patients might not fully represent the general population when it comes to age, trauma mechanism, smoking status, and comorbidities. Although as much data as possible concerning baseline characteristics were retrieved, recollection bias could be an issue. Retrospective analyses do not allow for standardized follow-ups and the monitoring of outcome measures, such as fixed-term radiographs and final clinical recovery. However, we will be able to provide an overview of a large period of time to show possible differences in the outcomes of the two historic cohorts. Furthermore, as Dancer’s fractures are relatively rare, it would take several years to include a sufficient number of cases prospectively. With the use of a retrospective design, it is possible to gain a first insight into the outcomes of patients with Dancer’s fractures within a reasonable time frame.

### Future Directions

If this retrospective study shows promising results, a multicenter randomized controlled trial will be designed. Due to the relatively low incidence of Dancer’s fractures, a multicenter design is preferred. The information gathered in our investigation can be used for grant applications, future ethical approval, and patient selection.

### Conclusions

If the study described in this protocol is performed, it will be the largest study on this topic so far and the first to compare nonoperative and operative treatments for Dancer’s fractures. Our analysis will show if there are any associations among chosen treatments, delayed or absent fracture healing, and indications for secondary surgical intervention, as well as complications and functional outcomes. Our findings might be used as a basis for formulating a subsequent study protocol that intends to prove the superiority of either treatment modality prospectively and help guide decision-making for individual patients based on their characteristics.

## Abbreviations

SF-12: 12-Item Short Form Survey
WMO: Wet medisch-wetenschappelijk onderzoek met mensen
